# Senescent Cell Depletion Through Targeting BCL-Family Proteins and Mitochondria

**DOI:** 10.3389/fphys.2020.593630

**Published:** 2020-12-01

**Authors:** Ying Fan, Jiaoqi Cheng, Huihong Zeng, Lijian Shao

**Affiliations:** ^1^Department of Occupational Health and Toxicology, Medical College of Nanchang University, Nanchang, China; ^2^Department of Histology and Embryology, Medical College of Nanchang University, Nanchang, China; ^3^Jiangxi Provincial Key Laboratory of Preventive Medicine, Nanchang University, Nanchang, China

**Keywords:** senescence, aging, senolytics, apoptosis, p16^INK4a^

## Abstract

Senescent cells with replicative arrest can be generated during genotoxic, oxidative, and oncogenic stress. Long-term retention of senescent cells in the body, which is attributed to highly expressed BCL-family proteins, chronically damages tissues mainly through a senescence-associated secretory phenotype (SASP). It has been documented that accumulation of senescent cells contributes to chronic diseases and aging-related diseases. Despite the fact that no unique marker is available to identify senescent cells, increased p16^INK4a^ expression has long been used as an *in vitro* and *in vivo* marker of senescent cells. We reviewed five existing p16^INK4a^ reporter mouse models to detect, isolate, and deplete senescent cells. Senescent cells express high levels of anti-apoptotic and pro-apoptotic genes compared to normal cells. Thus, disrupting the balance between anti-apoptotic and pro-apoptotic gene expression, such as ABT-263 and ABT-737, can activate the apoptotic signaling pathway and remove senescent cells. Mitochondrial abnormalities in senescent cells were also discussed, for example mitochondrial DNA mutation accumulation, dysfunctional mitophagy, and mitochondrial unfolded protein response (mtUPR). The mitochondrial-targeted tamoxifen, MitoTam, can efficiently remove senescent cells due to its inhibition of respiratory complex I and low expression of adenine nucleotide translocase-2 (ANT2) in senescent cells. Therefore, senescent cells can be removed by various strategies, which delays chronic and aging-related diseases and enhances lifespan and healthy conditions in the body.

## Introduction

In the 1960s, Hayflick documented that normal human fibroblasts could not eternally grow, with irreversible cell cycle arrest occurring at a certain point, which is also known as replicative senescence (Hayflick and Moorhead, 1961; Hayflick, 1965). During cell division, telomere length shortens, which contributes to replicative senescence (Olovnikov, 1973; Lange, 2005). It is well accepted that cellular senescence could also be induced by various stressors, such as oncogenic, oxidative, and genotoxic conditions (Hernandez-Segura et al., 2018; Magalhaes and Passos, 2018), which is called premature senescence. Premature senescence is not related to telomere shortening. Replicative and premature senescence have shared features, which include an enlarged and flattened shape, senescence-associated beta-galactosidase (SA-β-gal) positive staining, and increased p16^INK4a^ expression (Hernandez-Segura et al., 2018). Upregulated levels of p16^INK4a^ expression inhibits the formation of catalytically active cyclin D-CDK complexes. It subsequently inactivates cyclin E-CDK2 and cyclin A-CDK2. Inhibition of cyclin E-CDK2 and cyclin A-CDK2 blocks G1 to S-phase transition and results in a G1-phase cell-cycle arrest and cellular senescence (Sherr, 2000; Ortega et al., 2002). Therefore, p16^INK4a^, coexisting with increased SA-β-gal activity, is often used as a robust senescence marker in various mouse and human tissues.

Senescent cells avoid apoptosis and necrosis despite their high levels of metabolism, DNA damage, and senescence-associated secretory phenotype (SASP) (Dörr et al., 2013; Wiley and Campisi, 2016). Additionally, senescent cells, but not healthy and replicating cells, are resistant to harsh microenvironments, such as nutritionally depleted conditions (Sagiv et al., 2013). These characteristics keep them alive in the body for a long time, which might lead to chronic diseases and age-related dysfunction. It has been shown that an increased number of senescent cells in tissues is correlated to many age-related diseases, such as chronic pulmonary disease, chronic bone marrow injury, osteoarthritis, atherosclerosis, neurodegenerative disorders, chronic renal diseases, and diabetes (Childs et al., 2017; He and Sharpless, 2017; McHugh and Gil, 2018; Barnes et al., 2019; Docherty et al., 2019). Although senescent cells only occupy around 15% of dysfunctional tissues, the accumulation of senescent cells potentially causes chronic tissue dysfunction, promotes tissue destruction and aging, causes local inflammation, and can even lead to tumorigenesis and metastasis (Herbig et al., 2006). The negative effects associated with senescent cells might be attributed to SASP, indicating that these cells secrete various chemokines, pro-inflammatory cytokines, and extracellular matrix proteases (Coppé et al., 2008, 2010).

The existence of senescent cells has double-edged effects. On the one hand, the short-term appearance of senescent cells has a positive function in suppressing tumorigenesis during early stages (Kang et al., 2011; Baker et al., 2017; Calcinotto et al., 2019), accelerates wound healing (Adams, 2009; Demaria et al., 2014), and maintains tissue integrity during embryonic development (Munoz-Espin et al., 2013; Storer et al., 2013; Davaapil et al., 2017). On the other hand, long-term accumulation of senescent cells is deleterious to tissues through the SASP (Herranz and Gil, 2018; Gorgoulis et al., 2019). Senescent cells are usually removed by the immune system instead of apoptotic and necrotic mechanisms (Burton and Stolzing, 2018; Prata et al., 2018). However, senescent cells will not efficiently be cleared in various tissues if the capacity of the immune system is dysfunctional or if existing senescent cells exceed the clearing capacity of the immune system. Thus, we must develop efficient approaches, such as *in vivo* depletion of senescent cells, to rejuvenate tissue function.

It is necessary to understand how the existence of senescent cells is regulated and what underpins the molecular mechanisms of these cells in order to discover new strategies that allow selective depletion of senescent cells *in vitro* and *in vivo*. The clearance of senescent cells has been shown to delay and reduce various tissue aging phenotypes in premature and natural aged models, such as BubR1 and Ercc1 knockout progeroid mice (Niedernhofer et al., 2006; Baker et al., 2008, 2011; Childs et al., 2016). Efficient clearance will create good opportunities to assess the functional importance of cellular senescence in different pathological conditions, potentially leading to novel therapeutics. In this review, we discuss five different p16^INK4a^ reporter mice and various known pharmacological strategies to clear senescent cells. We also highlight the importance of targeting mitochondria in selectively depleting senescent cells. Underlying mechanisms of SASP in senescent cells was not discussed here as they have been extensively reviewed by others (Tchkonia et al., 2013; Gorgoulis et al., 2019).

## Monitoring and Clearing Senescent Cells

The importance of senescent cells in aging and age-related diseases motivated investigators to find a proper reporter system to monitor and identify senescent cells *in vitro* and *in vivo*. First, the essential senescent markers should be identified, as there is currently no common senescent marker in different cells or tissues. Hence, several different methods have to be used to properly define senescent cells. This includes: flattened and enlarged morphology (Munoz-Espin and Serrano, 2014; Simay et al., 2018); SA-β-gal positive staining (Dimri et al., 1995; Kurz et al., 2000); increased p16^INK4a^ expression (Krishnamurthy et al., 2004; Ressler et al., 2006; Liu et al., 2009); DNA damage (Sedelnikova et al., 2004; d’Adda di Fagagna, 2008; Hewitt et al., 2012); and SASP-related factors (Coppé et al., 2010; Tchkonia et al., 2013; Hernandez-Segura et al., 2018). Thus far, it is well accepted that the induction of p16^INK4a^ expression is a hallmark of cellular senescence in most scenarios. Currently, there are five different p16^INK4a^ reporter mice available, as discussed below (Table 1).

**Table 1:** Comparison of five p16^INK4a^ reporter mice models.

P16^INK4a^ reporter mice	Genetic strategy	Reporter	Features	Refs
P16-Luc	Transgenic	Luciferase	Monitoring senescent cells timely	Yamakoshi et al., 2009
P16^LUC^	Knockin	Luciferase	Monitoring senescent cells timely	Burd et al., 2013
P16^tdTom/+^ mice	Knockin	tdTomato	1. Isolating and analyzing single senescent cell 2. Fluorescence of tdTom is six times brighter than green fluorescent protein	Liu et al., 2019
INK-ATTAC mice	Transgenic	GFP, ATTAC	1. Isolating and analyzing single senescent cell 2. Clearing senescent cells by AP20187	Baker et al., 2011
P16-3MR mice	Transgenic	Luciferase, mRFP, HSV-TK	1. Monitoring senescent cells timely. 2. Isolating and analyzing single senescent cell 3. Clearing senescent cells by GCV	Demaria et al., 2014

### p16-Luc Transgenic Mouse

In 2009, Dr. Hara’s group used a genomic DNA segment containing the entire human INK4a/Arf gene locus to engineer a fusion protein with firefly luciferase (Luc, named p16-Luc) without deleting any genomic DNA sequences at the INK4a/Arf gene locus, generating transgenic p16-Luc mouse (Yamakoshi et al., 2009). Advantages of this model include: (1) p16-Luc fusion protein was used to specify p16^INK4a^ gene expression instead of p19^INK4d^ gene expression in their overlapping gene locus; (2) luciferase activity was used to monitor p16^INK4a^ expression timely; and (3) p16^INK4a^ family genes p15^INK4b^, p18^INK4c^, and p19^INK4d^ did not disrupt p16^INK4a^ gene expression in this reporter. Luciferase activity of mouse embryonic fibroblasts (MEFs) from p16-Luc mice was significantly increased by the induction of cellular senescence. Endogenous mouse p16^INK4a^ expression was also increased in senescent MEFs. These data imply that the expression of human p16^INK4a^ represents mouse cell senescence. Using the reporter, it confirmed that the increased p16^INK4a^ gene expression could inhibit tumorigenesis and was not derived from cell culture conditions in cultured cells (Gil and Peters, 2006; Ohtani et al., 2010). These results resolved the challenge of whether the increased p16^INK4a^ gene expression in tissue culture was a reflection of cellular senescent status and anti-cancer process.

To test the functional ability *in vivo*, they used naturally aging p16-Luc mice to measure luciferase activity. The data implies that the bioluminescent signaling level was dramatically increased in p16-Luc mice during natural aging. Importantly, they found that oncogenic Ras activation caused epigenetic derepression of p16^INK4a^ expression through reduction of DNA methyl transferase 1 (DNMT1) levels *in vivo* (Yamakoshi et al., 2009). By using a DMBA/TPA-induced papillomas mouse model, they proved that p16^INK4a^ upregulation could efficiently prevent malignant conversion of benign tumors. This is due to the reduction of histone 3 Lys9 demethylation (H3K9me2) in papillomas, which leads to increasing p16^INK4a^ expression. In the case of p53 mutation, the timeframe of p16^INK4a^ upregulation is much shorter. This indicates that p16^INK4a^ expression, as a backup tumor suppressor, is regulated by p53 activity (Yamakoshi et al., 2009). Therefore, cellular senescence is an anti-tumor defense mechanism prompting oncogenic cells into cell cycle arrest.

### p16^LUC^ Knockin Mouse

In 2013, Dr. Sharpless’s group exploited the highly dynamic induction of p16^INK4a^ observed in response to oncogenic insult and senescence. They used a “knock in” strategy to generate an *in vivo* reporter system, p16^LUC^ (Burd et al., 2013). The firefly luciferase cDNA followed by an SV40 polyadenylation signal was targeted into the translational start site of the endogenous p16^INK4a^ locus. In contrast to previous transgenic reporter systems driven by fragments of the p16^INK4a^ promoter, they preserve the known cis-regulatory elements centromeric to the p16^INK4a^ open reading frame by the “knock in” strategy. The p16^LUC^ allele was utilized to detect senescence and the earliest steps of tumorigenesis in well-defined murine systems.

To examine the correlation between p16^INK4a^ expression and age, p16^+/LUC^ mice at 16 weeks of age were used to monitor luciferase activity during the mouse natural aging process. The results showed that the luciferase activity was significantly increased during a period of 80 weeks, indicating that p16^LUC^ expression represents a faithful and age-related increase in p16^INK4a^ gene expression. It is well known that both wound healing and mammary involution models have high p16^INK4a^ gene expression (Gadd et al., 2001; Adams, 2009; Demaria et al., 2014). p16^LUC^ reporter in terms of wound healing and mammary involution models showed that increased luciferase activity was easily detected during both processes, while the activity was significantly reduced upon completion of wound healing and mammary involution. Senescent cells secrete platelet-derived growth factor AA (PDGF-AA) to induce myofibroblast differentiation, leading to wound closure. Consistently, application of recombinant PDGF-AA onto a wound area promotes its recovery by inducing myofibroblast differentiation (Demaria et al., 2014). These results indicate that cellular senescence has some beneficial roles during tissue repair by secreting SASP factors.

Importantly, the p16^LUC^ targeting strategy with an allele null for p16^INK4a^ does not affect expression of p15^INK4b^ and p19^INK4d^. The luciferase activity of p16^LUC^ both *in vitro* and *in vivo* robustly reflects endogenous p16^INK4a^ expression. In addition, previous studies have shown that p16^INK4a^ expression was remarkably increased in the early stage but not advanced stage of tumor development (Collado et al., 2007). p16^LUC^ reporter will provide an opportunity to detect early-stage tumors, monitor cancer progression, and test anti-cancer drugs. The data further confirms that p16^INK4a^ is a tumor suppressor, limiting cancer progression and cell proliferation. Therefore, p16^LUC^ reporter can be used to dynamically monitor cells undergoing senescent progression under physiological and pathological settings.

### p16^tdTom/+^ Knockin Mouse

As mentioned above, both p16-Luc and p16^LUC^ reporters have the ability to monitor senescent cells with p16^INK4a^ overexpression. However, both reporters cannot be used to isolate and analyze individual senescent cells based on luciferase activity. To this end, Dr. Sharpless’s group generated a murine p16^INK4a^ reporter mice by knocking in a tandem-dimer Tomato (tdTom) into exon 1a of the p16^INK4a^ locus in 2019 (Liu et al., 2019). They used the reporter line to enumerate, isolate, and characterize the individual p16^INK4a^-expressing tdTom^+^ cells. The frequencies of tdTom^+^ cells were increased with serial passage in cultured MEFs from p16^tdTom/+^ mice *in vitro*. They also used a peritoneal inflammation mice model to demonstrate that tdTom^+^ macrophages exhibited some features of senescence *in vivo*, which are reduced proliferation, SA-β-gal positive staining, and increased mRNA expression of a subset of transcripts encoding factors involved in SASP, such as MMP12 and Cxcl12 (Liu et al., 2019). These results indicate that cells harboring activation of the p16^INK4a^ promoter accumulate with aging and inflammation *in vivo*, and display characteristics of senescence. Moreover, tdTom fluorescence is six times brighter than green fluorescent protein (GFP) without the predisposition to aggregation and toxicity. Another feature in the p16^tdTom/+^ knockin reporter is that the expression of tdTom faithfully assesses not only p16^INK4a^ mRNA transcript abundance but also p16^INK4a^ promoter activation. The half-life of tdTom mRNA is about 12 h, whereas that of p16^INK4a^ and p19^INK4d^ mRNA is up to 24 h (Hara et al., 1996; Liu et al., 2019). p16^tdTom/+^ knockin reporter does not reflect those senescent cells with low p16^INK4a^ promoter activity. Thus, the reporter cannot detect all senescent cells *in vitro* and *in vivo*.

In comparison to p16-Luc and p16^LUC^ reporter mice, p16^tdTom/+^ reporter allows the detection and isolation of individual senescent cells that express bright tdTom fluorescence. Hence, p16^tdTom/+^ reporter is a perfect tool to perform downstream analysis in senescent cells, such as single cell RNA-Seq, or to find new senescent markers for future studies. p16-Luc mouse model from Dr. Hara’s group and p16^LUC^ mouse model from Dr. Sharpless’s group can be used for timely monitoring of senescent cell appearance *in vitro* and *in vivo*. Therefore, p16^tdTom/+^ mouse model provides a good opportunity to detect, isolate, and analyze single senescent cells. However, all three models do not have the ability to clear senescent cells *in vivo*.

### INK-ATTAC Transgenic Mouse

In 2011, Dr. van Deursen’s group developed a transgenic mouse model with features of both monitoring and clearing senescent cells in order to examine the role of cellular senescence in aging and age-related diseases (Baker et al., 2011). They designed a transgenic strategy of constructing FKBP-Caspase8-Flag fusion protein and EGFP under the control of a 2616-bp fragment of the p16^INK4a^ promoter (named INK-ATTAC mice). A synthetic drug AP20187 induces dimerization of a membrane-bound myristoylated FK506-binding protein-caspase 8 (FKBP-Casp8) fusion protein expressed specifically in senescent cells *via* the control of p16^INK4a^ promoter. BubR1^H/H^, INK-ATTAC mice were generated by crossing INK-ATTAC mice with BubR1 mice, which is a progeroid mouse model. GFP^+^ and GFP^−^ cell populations from inguinal adipose tissue in aged BubR1^H/H^, INK-ATTAC mice were separated to measure p16^INK4a^ expression. Expression of p16^INK4a^ were higher in GFP^+^ cells than GFP^−^ cells isolated from aged BubR1^H/H^, INK-ATTAC mice. In addition, GFP^+^ cells were also detected in primary MEFs with ectopic expression of oncogenic Ras or serial passaging to induce cellular senescence. Taken together, these results indicate that INK-ATTAC is selectively expressed in p16^INK4a^-positive senescent cells.

Elimination of p16^INK4a+^ cells from non-progeroid mice using ATTAC by AP20187 could be used to address how senescent cells influence health and lifespan (Baker et al., 2016). Interestingly, AP20187 treatment delayed cataract formation in both males and females with a C57BL/6 background and reduced glomerulosclerosis independent of sex and genetic background. Depletion of senescent cells is correlated with attenuated age-related blood urea nitrogen increase, which benefits kidney functional recovery (Baker et al., 2016). The negative effect of age-related accumulation of p16^INK4a+^ cells was evidenced by the observation of lifespan extension in AP20187-treated INK-ATTAC mice (Baker et al., 2016).

The healthy condition of transgenic INK-ATTAC mice can be enhanced by selectively killing senescent cells with AP20187. The accumulation of senescent cells in association with several diseases, disabilities, and chronological aging is likely to contribute to the causation and pathophysiology of these problems or their symptoms (Childs et al., 2015, 2017). Together with chronic, sterile inflammation, macromolecular dysfunction, and stem and progenitor cell dysfunction, cellular senescence may contribute to both aging phenotypes and increased susceptibility to a range of chronic diseases (Tchkonia and Kirkland, 2018). The clearance of senescent cells has shown beneficial effects *in vivo*, such as slowing the development of kyphosis, cataracts, and lipodystrophy in progeroid mice (Baker et al., 2011).

The INK-ATTAC mice were also used to examine the effects of removing senescent cells on bone loss, aged cardiac deterioration, aged pancreatic β-cells, and other pathological conditions (Farr et al., 2017; Aguayo-Mazzucato et al., 2019; Lewis-McDougall et al., 2019). Elimination of senescent cells with AP20187 treatment benefits the increment of bone mass, activation of resident cardiac progenitor cells, and improvement of pancreatic β-cell function. These beneficial effects of depleting senescent cells contribute to the decrease of SASP program and expression of senescent markers. Compared to p16-Luc and p16^LUC^ mouse models, the INK-ATTAC mouse model has the notable advantage of clearing senescent GFP^+^ senescent cells. However, the mouse model fails to timely monitor senescent cells through luciferase activity in living animals.

### p16-3MR Transgenic Mouse

In 2014, Dr. Campisi’s group developed a reporter mouse with three features: monitoring, isolating, and depleting senescent cells (Demaria et al., 2014). They generated transgenic p16-3MR mice, which contain functional domains of a synthetic Renilla luciferase (LUC), monomeric red fluorescent protein (mRFP), and truncated herpes simplex virus 1 (HSV-1) thymidine kinase (HSV-TK) under the control of p16^INK4a^ promoter (Demaria et al., 2014). LUC allows the detection of 3MR-expressing cells by luminescence both *in vitro* and *in vivo*. mRFP allows the isolation of senescent cells from tissues. HSV-TK allows for the killing effects of ganciclovir (GCV), a nucleoside analog that has a high affinity for HSV-TK but a low affinity for the cellular TK. HSV-TK converts GCV into a toxic DNA chain terminator in nondividing senescent cells. GCV fragments mitochondrial DNA, causing death by apoptosis (Laberge et al., 2013).

The 3MR transgene activity was confirmed using MEFs from p16-3MR mice, which were induced to undergo senescence after irradiation (Demaria et al., 2014). Cellular senescence was verified by SA-β-gal positive staining and increased expression of p16^INK4a^, p21^Cip1^, and SASP factors. Senescent p16-3MR MEFs, compared to non-senescent cells, have higher levels of luciferase activity with mRFP expression shown *via* immunostaining. Moreover, GCV selectively removed senescent p16-3MR MEFs without toxicity on non-senescent p16-3MR MEFs. Increased luciferase activity was observed in living animals and different organs such as visceral fat, kidneys, and lungs at 3 months after 7.0 Gy irradiation (Demaria et al., 2014). The expression of p16, mRFP, and SASP factors in irradiated organs was significantly increased. GCV treatment in irradiated p16-3MR mice markedly reduced total body luminescence and senescence-associated gene expression in fat, kidneys, and lungs. In naturally aged mice model, p16-3MR mice showed a significant increase in luciferase activity starting at 18 months of age. GCV treatment in 20–24-month-old mice efficiently eliminated senescent cells determined by reduced luminescence, SA-β-gal positive staining, and p16^INKk4a^ expression in visceral fat (Demaria et al., 2014). Thus, these findings show that p16-3MR transgenic mice represent the presence of senescent cells through luminescence assay and allow the removal of senescent cells *via* GCV treatment.

Currently, the p16-3MR mouse model is largely used to explore the effects of senescence clearance under different pathological settings. Jeon et al. found that senescent cells were present in articular cartilage and synovium in osteoarthritis mouse model (Jeon et al., 2017). Clearance of these senescent cells *via* GCV treatment significantly increased cartilage development and pain reduction, which attenuates osteoarthritis development (Jeon et al., 2017). In intervertebral degeneration (IDD) model of p16-3MR mice, Patil et al. demonstrated that luciferase activity was high in the intervertebral discs with increasing SASP, which might be attributed to the development of IDD (Patil et al., 2019). Elimination of senescent cells by GCV treatment significantly decreased senescent cells with reducing SASP and disc aggrecan proteolytic degradation, increasing proteoglycan matrix and improving histological disc features (Patil et al., 2019). We irradiated p16-3MR mice to induce hematopoietic stem cell senescence with bone marrow suppression (Chang et al., 2016). GCV treatment could significantly decrease senescent hematopoietic stem cells, which is consistent with increasing hematopoietic stem cell engraftment by bone marrow transplantation assay. We also found that GCV treatment balanced lymphoid and myeloid cell differentiation from hematopoietic stem cells in irradiated mice. These data further confirm that hematopoietic stem cell senescence is a crucial mediator in irradiation-induced long-term bone suppression.

Overall, there are advantages and disadvantages in the five different p16^INK4a^ reporter mice. We can use p16-Luc, p16^LUC^, and p16-3MR mice to timely monitor senescent cells because of existing luciferase in these reporters. However, it might be a challenge to isolate single p16^INK4a^ positive cells due to the weak fluorochrome in INK-ATTAC and p16-3MR reporters. p16^tdTom/+^ reporter has the brightest fluorochrome when compared to INK-ATTAC and p16-3MR reporters. Therefore, p16^tdTom/+^ reporter might be the best option to isolate and analyze single senescent cells to perform downstream analysis. To deplete senescent cells *in vitro* and *in vivo*, both INK-ATTAC and p16-3MR mouse models can be chosen while p16-Luc, p16^LUC^, and p16^tdTom/+^ reporters cannot be used for the clearance of senescent cells.

## Expression of Apoptotic-Related Genes and Senescent Cell Clearance

It is well accepted that senescent cells can be monitored and depleted using five different p16^INK4a^ reporter mice, mentioned above. From a therapeutic point of view, it would be better to develop applicable strategies to deplete senescent cells to counter age-related diseases. To reach that point, we must explore the molecular properties of senescent cells. Here, we mainly discussed expression of apoptotic-related genes in senescent cells, which benefits the development of mechanism-based senescent cell clearance.

### Expression of BCL-Family Protein in Senescent Cells

**Figure 1:**
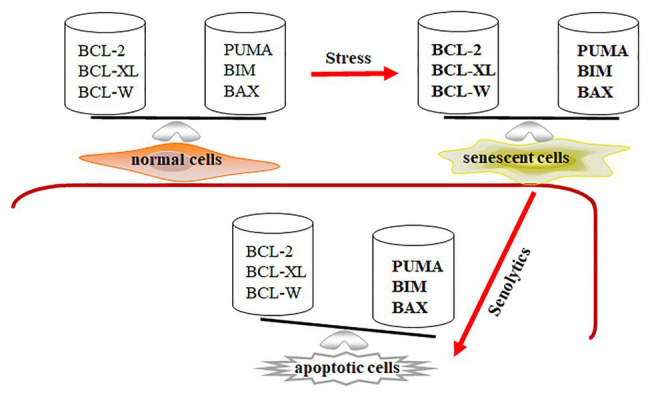
Senescent cells are in a primed apoptotic status. In normal cells, the expression of anti-apoptotic and pro-apoptotic genes is in a well-balanced condition. In senescent cells, the expression of anti-apoptotic and pro-apoptotic genes is abnormally upregulated (expressed in bold) and temporarily balanced, leading to primed apoptotic status for senescent cells. Decreasing expression of Bcl-2, Bcl-xl, and Bcl-w by senolytics disrupts the balance in senescent cells, leading to apoptotic cell death.

As we mentioned above, senescent cells are more resistant to apoptosis and harsh environments, leading to their retention within tissues. Expression of Bcl-2, Bcl-w, and Bcl-xl was significantly increased in senescent IMR90 cells induced by irradiation, Ras over-expression, and replication (Yosef et al., 2016). Through ribosome sucrose gradient fractionation analysis, the increase of Bcl-xl expression in senescent cells is shown to be due to the increased translational rates of Bcl-xl in irradiation-induced senescent cells. Baar et al. used senescent IMR90 cells to show that expression of pro-apoptotic genes Puma and Bim were significantly upregulated (Baar et al., 2017). We also demonstrated that senescent WI-38 cells had properties with increasing expressions of pro-apoptotic Bak and anti-apoptotic Bcl-xl (Chang et al., 2016). Therefore, BCL-family proteins are an essential component of senescent cells’ ability to be retained in the tissue. Expression of anti-apoptotic and pro-apoptotic genes is balanced at higher levels in senescent cells compared to that in normal cells. The profile of Bcl-family gene expression suggests that senescent cells might be primed to undergo apoptosis, while the execution of the death program is temporarily suppressed (Figure 1).

Knockdown of BCL-family protein was applied to senescent cells, showing that senescent cells are sensitive to downregulation of Bcl-xl, Bcl-w, and Bcl-2 (Zhu et al., 2016). Through siRNA or shRNA techniques, the downregulation of both Bcl-xl and Bcl-w could selectively remove IMR90 and WI-38 senescent cells (Chang et al., 2016; Yosef et al., 2016). However, the downregulation of either Bcl-xl or Bcl-w failed to deplete senescent cells. ABT-199, a Bcl-2 inhibitor, could not clear senescent cells. Combination of ABT-199 and siRNAs against Bcl-xl and/or Bcl-w significantly reduced the viability of senescent cells. Using pan-caspase inhibitors z-VAD-FMK or Q-VD-OPH, the depletion effects of knocking down Bcl-xl and Bcl-w on senescent cells were blocked (Chang et al., 2016; Yosef et al., 2016), indicating that the downregulation of Bcl-xl and Bcl-w reduces senescent cell viability *via* cellular apoptosis. Therefore, expression of Bcl-xl, Bcl-w, and Bcl-2 contributes to the resistance of senescent cells to apoptosis. The data also suggests that inhibitors that can block Bcl-xl, Bcl-w, and Bcl-2, which may help clear senescent cells.

### Inhibition of Bcl-2 Family Protein Sensitizes Senescent Cells

It has been shown that Bcl-xl, Bcl-w, and Bcl-2 inhibitors ABT-263 and ABT-737 could selectively clear senescent cells by more than 65% (Table 2), and not negatively affect normal cells (Chang et al., 2016; Yosef et al., 2016; Zhu et al., 2016). Both ABT-737 and ABT-263 treatment significantly reduced expression of Bcl-xl and Bcl-w in irradiation-induced lung fibrosis mouse models, which in turn decreased the numbers of senescent cells and ameliorated lung fibrosis (Pan et al., 2017). In double-transgenic K5-rtTA/tet-p14 mice, the induction of p14^Arf^ expression accelerates the generation of senescent epidermal cells, which can be cleared by ABT-737 treatment (Yosef et al., 2016). Administration of ABT-737 in K5-rtTA/tet-p14 mice benefits hair follicle stem cell proliferation, which repopulates the stem cell compartment in the bulge. We also showed that ABT-263 treatment in irradiated mice efficiently removed senescent hematopoietic and smooth muscle stem cells by increasing the stem cell’s functions of self-renewal and differentiation (Chang et al., 2016). These positive effects of removing senescent stem cells on stem cells were also confirmed using p16-3MR mouse model (Chang et al., 2016). Bussian et al. used MAPT^P301S^ PS19 mouse model of tau-dependent neurodegenerative disease to investigate whether the depletion of senescent cells *via* ABT-263 ameliorates the tau-dependent pathology while preserving cognitive function (Bussian et al., 2018). Their data showed that administration of ABT-263 starting at weaning age prevented the upregulation of senescence-associated genes with attenuating tau phosphorylation in PS19 mice. While studying the effects of senescent Osx1^+^ osteoprogenitors on bone loss with age, it was shown that ABT-263 had the ability to clear senescent Osx1^+^ cells with increasing osteoblasts and decreasing osteoclast formation (Kim et al., 2017). Additionally, Bcl-xl inhibitors, A1331852 and A1155463, can efficiently increase caspase 3/7 activity and cellular apoptosis in senescent HUVEC and IMR90 cells (Zhu et al., 2017). The data indicates that therapeutic targeting senescent cells by senolytics can ameliorate senescent cell-mediated pathological injury. This may also suggest that senescent cells negatively affect chronic and age-related diseases.

**Table 2:** Clearance of senescent cells by various senolytics.

Senolytic agents	Senolytic targets	Killing senescent cells	Refs
Dasatinib + Quercetin	EFNB, PI3K, P21^Cip1^, PAI2, Bcl-xl	Pre-adipocytes, HUVECs, MEFs, BM-MSCs	Zhu et al., 2015
ABT-263	Bcl-2, Bcl-xl, Bcl-w	WI-38, IMR-90, HRECs, MEFs, HUVECs, HSCs, MuSCs	Chang et al., 2016; Zhu et al., 2016
ABT-737	Bcl-2, Bcl-xl, Bcl-w	IMR-90, MEFs	Yosef et al., 2016
UBX0101	MDM2-P53	Chondrocytes	Jeon et al., 2017
FOXO4-DRI	FOXO4-P53	IMR-90, WI-38, BJ	Baar et al., 2017
17-DMAG	HSP90-AKT	MEFs, MSCs, IMR-90, WI-38	Fuhrmann-Stroissnigg et al., 2017
EF24	Bcl-xl, Mcl-1	WI-38, IMR-90, HUVECs, HRECs, pre-adipocytes	Li et al., 2019
Piperlongumine (PL)	Oxidation resistance 1	WI-38	Wang et al., 2016; Zhang et al., 2018
Fisetin	PI3K/ AKT	HUVECs	Zhu et al., 2017
A1331852, A1155463	Bcl-xl	HUVECs, IMR-90	Zhu et al., 2017
2-deoxy-D-glucose (2-DG)	Glycolysis	CISCs(lymphomas), vascular smooth muscle cells	Dörr et al., 2013; Gardner et al., 2015
Panobinostat (pano)	Histone 3 acetylation, Bcl-xl	CISCs (NSCLC, HNSCC)	Samaraweera et al., 2017
PZ15227	Bcl-xl, E3 ligase cereblon	WI-38, HREC, pre-adipocytes	He et al., 2020b

EFNB, ephrin ligand (EFN) B1 and B3; PI3K, phosphatidylinositol-4, 5-bisphosphate 3-kinase; PAI-2, plasminogen-activated inhibitor-2; HSP90, heat shock protein 90; HUVECs, human umbilical vein epithelial cells; MEFs, murine embryonic fibroblasts; BM-MSCs, bone marrow-derived murine mesenchymal stem cells; HRECs, human renal epithelial cells; WI-38, human lung fibroblasts; IMR-90, human lung fibroblasts; HSCs, hematopoietic stem cells; MuSCs, muscle stem cells; BJ, human foreskin fibroblasts; MSCs, murine mesenchymal stem cells; CISCs, chemotherapy-induced senescent cells; NSCLC, non-small cell lung cancer; HNSCC, head and neck squamous cell carcinoma.

Because ABT-263 has platelet toxicity that limits its further application, Gonzalez-Gualda et al. recently used galactose-conjugation of ABT263 to selectively induce senescent cell apoptosis without thrombocytopenia, which enhances the cytotoxicity of cisplatin in human A549 lung cancer cells (González-Gualda et al., 2020). To reduce the thrombocytopenia induced by ABT-263, Khan et al. re-constructed ABT-263 to DT2216, targeting Bcl-xl (Khan et al., 2019). They showed that DT2216 inhibited leukemia cell growth without platelet toxicity. However, the effectiveness of DT2216 on senescent cells remains unknown. Most recently, Dr. Zhou’s group used proteolysis-targeting chimera technology to develop another ABT-263 mimic, PZ15227, which could selectively remove senescent cells while improving the function of tissue stem cells in naturally aged mice (He et al., 2020b). Moreover, PZ15227 does not have an *in vivo* thrombocytopenia effect. Therefore, it should be further tested whether PZ15227 can improve tissue function by clearing senescent cells in different aged models, such as lung fibrosis or radiation-induced chronic bone marrow damage (He et al., 2020b).

### Combination of Dasatinib and Quercetin Sensitizes Senescent Cells

To investigate different approaches of removing senescent cells, Zhu et al. used transcriptional analysis using normal and senescent cells and found the increased expression of pro-survival genes, which maintains senescent cells’ resistance to apoptosis (Zhu et al., 2015). Combination of dasatinib (D) and quercetin (Q) was utilized to eliminate senescent human endothelial cells, mouse bone marrow-derived mesenchymal stem cells, and fat progenitors by reducing expression of p21^Cip1^, PAI-2, and Bcl-xl in senescent cells (Table 2). Similarly, fisetin, a quercetin analog, selectively removes senescent HUVEC cells by activating the apoptotic signaling pathway (Zhu et al., 2017). Using Ercc1^−/−^ mice as an accelerated aging model, Zhu et al. treated these aged mice with D + Q to delete senescent cells *in vivo* (Zhu et al., 2015). Their data demonstrate that periodic treatment with D + Q senolytics is sufficient to reduce senescence cells along with decreasing frailty and extending lifespan significantly. D + Q senolytic agents enhanced cardiac and vascular function in aging mice, reduced dysfunction caused by localized irradiation, and alleviated skeletal and neurological phenotypes in progeroid mice (Zhu et al., 2015).

In idiopathic pulmonary fibrosis (IPF), fibrogenic senescent fibroblasts were selectively killed by a senolytic cocktail, D + Q (Schafer et al., 2017). It was demonstrated that early ablation of senescent cells improved pulmonary function and physical health in the bleomycin-injury IPF model, although lung fibrosis is visibly unaltered. Using D + Q treatment, chronic clearance of senescent cells improves established vascular phenotypes associated with aging and chronic hypercholesterolemia (Roos et al., 2016). Removing senescent cells may be a viable therapeutic intervention to reduce morbidity and mortality from cardiovascular diseases (Roos et al., 2016). D + Q treatment resulted in significant reduction of senescent cells in the medial layer of aorta from aged and hypercholesterolemic mice, but not in intimal atherosclerotic plaques (Roos et al., 2016). Collectively, this study shows that chronic pharmacological clearance of senescent cells alleviates vasomotor dysfunction in naturally aged mice as well as in mice with atherosclerosis. Furthermore, senescent cell clearance reduces osteogenesis in advanced intimal plaques, ultimately reducing intimal plaque calcification. Therefore, the combination of D + Q attenuates the negative effects of senescent cells in aged and progeroid mice.

### Regulating p53 Levels and Senescent Cell Clearance

**Figure 2:**
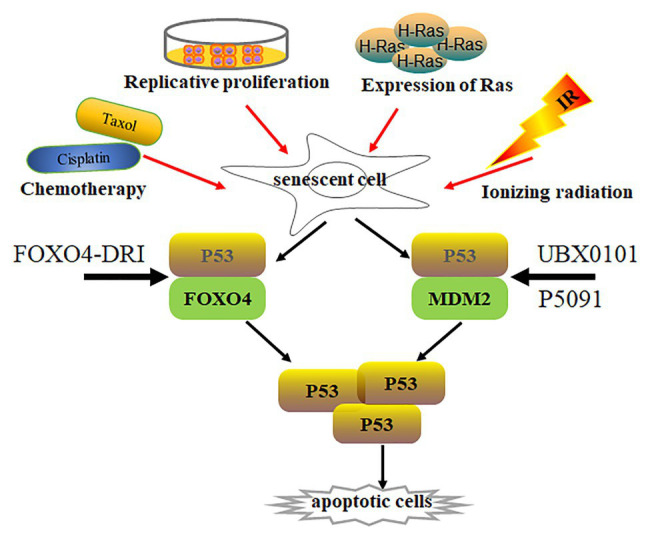
Senescent cell clearance *via* increasing p53 activity. Various situations, such as chemotherapy (Cisplatin, Taxol), radiation, replicative proliferation, and H-Ras overexpression, induce normal cells to undergo senescence. Activity of p53 is lower in most senescent cells than in normal cells. p53 forms a complex with Foxo4 or Mdm2, regulating p53 stabilization. Disruption of p53/Foxo4 or p53/Mdm2 interaction increases p53 activity in senescent cells through Foxo4-DRI or UBX0101 and P5091 treatment, respectively. Increasing p53 activity promotes senescent cell apoptosis.

Irradiation‐ and oncogene-induced senescence are attributed to DNA damage and subsequently increase p53 expression and its activity (Sherr, 2006; Cheng and Chen, 2010; Purvis et al., 2012) (Figure 2). p53 activation not only increases pro-apoptotic gene expression with cellular apoptosis, but also leads to p21^Cip1^ upregulation and initiates cellular senescence (Karimian et al., 2016; Hafner et al., 2019). p21^Cip1^ overexpression promotes cell cycle arrest along with consecutive p16^INK4a^ upregulation. However, p53 levels are much lower in senescent cells than those in normal cells (Kim et al., 2015; Johmura and Nakanishi, 2016), indicating that p53 activation is dispensable in senescent cells. To investigate how p53 expression was regulated in senescent cells, Sisoula et al. showed that chaperone-associated ligase CHIP was highly expressed in senescent cells, promoting p53 degradation below the basal level (Sisoula et al., 2011). Accordingly, p53 degradation was observed by the inhibition of HSP90 activity in senescent cells but not in normal cells (Sisoula et al., 2011). Johmura et al. demonstrated that SCF (Fbxo22) was highly expressed in senescent cells and formed a complex with a lysine demethylase KDM4A, which can target methylated p53, regulating p53 degradation in senescent cells (Johmura et al., 2016).

p53 is a well-known tumor suppressor that modulates cellular senescence, cancer, and aging (Hasty and Christy, 2013; Rufini et al., 2013). Is there any special functional meaning of low p53 levels in senescent cells? Whether low p53 expression accelerates senescent cell transformation remains elusive. On the other hand, could p53 re-activation in senescent cells resume cellular apoptotic program by increasing pro-apoptotic gene expression? An opening question for this point is how to re-activate p53 in senescent cells. Recently, Baar et al. found that Foxo4 expression was increased in senescent cells, which might promote senescent cell survival by interacting with p53. The interaction inhibits p53-mediated cellular apoptosis (Baar et al., 2017). The peptide Foxo4-DRI can promote nuclear exclusion of active pSer15-p53 and decrease p21^Cip1^ expression, activating downstream target gene expression and inducing cellular-intrinsic apoptosis by caspase 3 cleavage. This resulted in clearing senescent IMR90 cells by activating the intrinsic apoptotic pathway without affecting normal cells. Foxo4-DRI also reduces *in vivo* doxorubicin-induced senescence and liver toxicity (Baar et al., 2017). The beneficial effects of Foxo4-DRI were also observed in fast-aging Xpd^TT/TTD^ and naturally aged mice (Baar et al., 2017). The Foxo4-DRI treatment improved fur density, renal function, and physical activity in aged mice. These data prove that increasing p53 activity can selectively remove senescent cells. However, Foxo4-DRI has a short half-life *in vivo* (Baar et al., 2017). Thus, different strategies to increase p53 activity must be investigated to remove senescent cells.

To activate intracellular p53, Jeon utilized UBX0101, a senolytic MDM2/p53 interaction inhibitor, to increase p53 activity and stability through blocking MDM2-mediated p53 ubiquitination and proteasome degradation (Jeon et al., 2017). UBX0101 can remove senescent cells *in vitro* and *in vivo*, resulting in alleviating post-traumatic osteoarthritis, reducing pain, and increasing cartilage development. Therefore, blocking the interaction between MDM2 and p53 can prevent p53 degradation, which increases p53 activity and deplete senescent cells. Most recently, He et al. demonstrated that ubiquitin-specific peptidase 7 (USP7) inhibitor P5091 accelerated MDM2 ubiquitination and degradation, which in turn increased intracellular p53 expression and cleared senescent cells by activating cellular apoptosis (He et al., 2020a). The depletion of senescent cells by P5091 alleviated doxorubicin-induced SASP and renal toxicities in mice. Therefore, increasing p53 activity in senescent cells is a novel senolytic strategy, which can be achieved by disrupting MDM2/P53 interaction to regulate the stability of p53 and MDM2.

### Other Pro-apoptotic Strategies Sensitize Senescent Cells

Since D + Q and ABT-263 can remove senescent cells, many other senolytics have been developed to selectively clear senescent cells. It has been reported that heat shock protein 90 (HSP90) inhibitor 17-DMAG (alvespimycin) eliminated senescent Ercc1^−/−^ MEFs through inducing apoptosis by inhibiting HSP90 and downregulating the anti-apoptotic PI3K/AKT pathway *in vitro* (Table 2). 17-DMAG treatment delays the onset of several age-related symptoms, and extends lifespan in Ercc1^−/−^ mice (Fuhrmann-Stroissnigg et al., 2017). EF24, a curcumin analog, selectively depletes senescent cells *via* cellular apoptotic mechanisms (Li et al., 2019). The killing effects of EF24 on senescent cells are dependent on the decreased expression of Bcl-xl and Mcl-1 *via* proteasome degradation, which induces cellular apoptosis. However, whether EF24 can remove senescent cells *in vivo* remains to be determined. Piperlongumine (PL) isolated from genus piper preferentially clears senescent human WI-38 fibroblasts through inducing cellular apoptosis but not ROS production (Wang et al., 2016). PL can directly bind to oxidation resistance 1 (OXR1), an important antioxidant protein that regulates the expression of a variety of antioxidant enzymes. PL selectively induces OXR1 degradation through the ubiquitin-proteasome system in a senescent cell-specific manner (Zhang et al., 2018).

2-deoxy-D-glucose (2-DG), an inhibitor of glycolysis, eliminates therapy-induced senescent cells *in vivo* and *in vitro* through caspase-12‐ and caspase-3-mediated endoplasmic reticulum-related apoptosis (Dörr et al., 2013). Furthermore, 2-DG leads to significant death of senescent vascular smooth muscle cells *in vitro* through apoptosis (Gardner et al., 2015). Panobinostat is a histone deacetylase inhibitor, which can significantly clear chemotherapy-induced senescent cells by increasing caspase 3/7 activity, decreasing expression of Bcl-xl, and increasing H3 acetylation in non-small cell lung cancer and head and neck squamous cell carcinoma cell lines (Samaraweera et al., 2017). Taken together, the killing mechanisms of current available senolytics are mainly through cellular apoptosis. However, these senolytics can remove around 65% of senescent cells through activating the intrinsic apoptotic pathway. These data suggest that other mechanism-based killing approaches of senescent cells might exist and be developed in the future. Thus far, it has not been extensively investigated whether expression of extrinsic apoptotic signaling-related proteins is changed in senescent cells. Does activation of the extrinsic apoptotic signaling pathway have the ability to clear senescent cells? Activation of both intrinsic and extrinsic apoptotic signaling pathways might efficiently remove senescent cells. These questions should be further investigated.

## Targeting Mitochondria and Clearance of Senescent Cells

As we reviewed above, both ABT-737 and ABT-263 have senolytic functions on senescent cells, which is mediated by the activation of the intrinsic mitochondrial-related apoptotic pathway. However, senescent cells have robust metabolic activity with increasing oxygen consumption, mitochondrial potential, energy, and ROS production. Many studies have shown the abnormal changes of mitochondria on senescent cells. Accumulation of mitochondrial DNA (mtDNA) mutation has been implicated in age-related diseases (Hahn and Zuryn, 2019). The phenomenon is supported in mitochondrial DNA polymerase gamma (PolG) mutant mice, showing that PolG mutant mice had premature aging phenotype with mtDNA mutation accumulation (Graziewicz et al., 2007). mtDNA mutation accumulation can drive electron transport chain (ETC) dysfunction and ROS production in senescent cells, forming a “vicious cycle” and enhancing mitochondrial damage (Hahn and Zuryn, 2019). Pharmacologic inhibition of ETC complexes has been reported to induce oxidative stress and premature senescence in cells (Stockl et al., 2006).

Mitophagy is essential for the clearance of damaged or dysfunctional mitochondria and prevention of cellular senescence. Mitophagy is regulated by mitochondrial fission and fusion. Impaired mitochondrial fission and fusion processes negatively affect cells’ ability to degrade dysfunctional mitochondria, causing ROS-induced DNA damage and senescence. Decreased Pink1 expression has detrimental effects on mitophagy and reduces the mitochondrial membrane potential along with mitochondrial dysfunction (Tsujimoto et al., 2020). Increasing mitophagy by activation of Pink1/Parkin pathway significantly decreases numbers of SA-β-gal positive cells in bronchial epithelial cells and mesenchymal stem cells (Tsujimoto et al., 2020). In addition, mitochondrial unfolded protein response (mtUPR) is initiated by cleavage of unfolded mitochondrial proteins into peptides by the protease CLPP-1. Those peptides are exported into the cytoplasm by the transporter HAF-1 (Shpilka and Haynes, 2018). Previous studies showed that dysfunction of mtUPR may play an important role in age-related pathology, which is supported by the extending lifespan under mtUPR activation conditions (Shpilka and Haynes, 2018). It is not clear whether activation of mtUPR is either sufficient for lifespan extension or necessary for lifespan extension in conditions where mitochondrial function is modulated, including caloric restriction. Other data also support that mtUPR has been shown to be necessary for enhanced survival in response to various stresses (Mao et al., 2016).

Because senescent cells have dysfunctional mitochondria with high metabolical activity, is it possible to specifically target mitochondria to remove senescent cells? Most recently, Neuzil’s group investigated the role of the chemical mitochondria-targeted tamoxifen (MitoTam) on depleting senescent cells (Hubackova et al., 2019a,b). MitoTam can specifically target respiratory complex I in the mitochondrial inner membrane by interfering with electron transfer, leading to ROS production. MitoTam treatment selectively removed senescent cells from cancer and normal tissues/cells, including human RPE-1, lung fibroblast HFP-1 and foreskin fibroblast BJ cells. Moreover, MitoTam selectively kills senescent cells both *in vitro* and *in vivo* through suppressing OXPHOS and decreasing mitochondrial potential independent of increasing ROS production. The clearance efficiency of MitoTam on senescent cells is four times greater than that of ABT-737. However, other complex I inhibitors, like IACS-010759 and metformin, failed to clear senescent cells (Hubackova et al., 2019a). This data indicates that targeting respiratory complex I is not the only mechanism of MtioTam on removing senescent cells.

Further data found that senescent cells expressed lower levels of adenine nucleotide translocase-2 (ANT2) when compared to normal cells. ANT2, as a mitochondrial carrier family protein, transfers ATP generated by glycolysis into mitochondria (Fiore et al., 1998). MitoTam selectively depletes senescent cells because of downregulation of ANT2. The sensitivity of senescent cells to MitoTam treatment can be counteracted by upregulation of ANT2. Consistently, the combination of complex I inhibitor rotenone and mitochondrial uncoupler CCCP selectively causes senescent cell death (Hubackova et al., 2019a). Simultaneous interference with mitochondrial structure and integrity, and ATP hemostasis in senescent cells, leads to their effective removal. Therefore, the interaction between ANT2 and ATP synthase plays a critical role in the maintenance of mitochondrial integrity. Targeting dysfunctional mitochondria by MitoTam opens a novel avenue to remove senescent cells in senescent-related diseases and pathologies.

## Discussion

We have discussed five different p16^INK4a^ reporters that were used to investigate senescent cells. However, some senescent cells do not highly express p16^INK4a^, and p21^Cip1^ upregulation was observed in senescent cells (Herbig et al., 2004). Therefore, p16^INK4a^ reporters might underestimate the actual numbers of senescent cells. It would be helpful if we could find other reporters to compensate p16^INK4a^ reporters to accurately assess senescent cells. For senescent cell clearance, INK-ATTAC or p16-3MR reporters only remove senescent cells with p16^INK4a^ upregulation and fail to deplete those senescent cells with low levels of p16^INK4a^ expression. Therefore, INK-ATTAC or p16-3MR reporters are not sufficient to clear all senescent cells in progeroid mice. Other reporters for senescent cells should be developed to better eliminate senescent cells. It is well known that p21^Cip1^ expression is increased in some senescent cells. Therefore, a p21^Cip1^ reporter mouse might be a good option to label or remove senescent cells. A combination of p21^Cip1^ reporter and INK-ATTAC or p16-3MR reporters may detect all senescent cells. This hypothesis needs to be tested in the near future.

Several senolytics have been developed to target senescent cells, but many of them have cell type specific killing effects. For example, senescent human pre-adipocytes are not sensitive to ABT-263 (Zhu et al., 2016), although ABT-263 kills more than 60% of senescent WI-38, IMR-90, HUVECs, and MEFs in 72 h (Chang et al., 2016). Fisetin could not eliminate senescent IMR-90 and preadipocytes, but efficiently cleared senescent HUVECs. A1331852 and A1155463 are senolytics in HUVECs and IMR-90 cells but not primary human preadipocytes (Zhu et al., 2017). Thus, it would be possible to target a specific type of senescent cells by senolytic designing. Another possible method is to combine different senolytics to clear more senescent cells. These approaches should be tested in further studies. As we discussed above, around 15% of cells with senescent features exist in aged tissues. The function of those senescent cells might contribute to the maintenance of tissue integrity when tissues are damaged. This opens the question of whether we should remove all senescent cells or part of them to keep tissue integrity, which should be investigated in the near future as well.

It is well-known that organelle homeostasis is crucial to maintain normal cellular function. Senescent cells have multiple functional defects in mitochondria, such as impaired mitochondrial morphology, mtDNA mutation, mitophagy, and mtUPR. Through targeting mitochondria, MitoTam can efficiently remove senescent cells with low levels of ANT2 protein expression. Along with dysfunctional mitochondria, other cellular organelles have abnormal functions in senescent cells, such as an unfolded protein response in the endoplasmic reticulum, an increase in senescence-associated-β-galactosidase activity in lysosome, senescence-associated heterochromatin foci, and DNA damage response in nucleus (Hwang et al., 2009). To efficiently deplete senescent cells, these dysfunctional organelles might become perfect candidates to ameliorate aging-related diseases in the future.

## Author Contributions

YF and LS designed the study. YF, JC, HZ, and LS drafted the manuscript. All authors contributed to the article and approved the submitted version.

### Conflict of Interest

The authors declare that the research was conducted in the absence of any commercial or financial relationships that could be construed as a potential conflict of interest.
